# Relation path feature embedding based convolutional neural network method for drug discovery

**DOI:** 10.1186/s12911-019-0764-5

**Published:** 2019-04-09

**Authors:** Di Zhao, Jian Wang, Shengtian Sang, Hongfei Lin, Jiabin Wen, Chunmei Yang

**Affiliations:** 1 School of Computer Science and Technology, Dalian University of Technology, Dalian, China; 2 Department of VIP, the Second Hospital of Dalian Medical University, Dalian, China

**Keywords:** Literature-based discovery, Drug discovery, Knowledge graph, Path ranking algorithm, Convolutional neural network

## Abstract

**Background:**

Drug development is an expensive and time-consuming process. Literature-based discovery has played a critical role in drug development and may be a supplementary method to help scientists speed up the discovery of drugs.

**Methods:**

Here, we propose a relation path features embedding based convolutional neural network model with attention mechanism for drug discovery from literature, which we denote as PACNN. First, we use predications from biomedical abstracts to construct a biomedical knowledge graph, and then apply a path ranking algorithm to extract drug-disease relation path features on the biomedical knowledge graph. After that, we use these drug-disease relation features to train a convolutional neural network model which combined with the attention mechanism. Finally, we employ the trained models to mine drugs for treating diseases.

**Results:**

The experiment shows that the proposed model achieved promising results, comparing to several random walk algorithms.

**Conclusions:**

In this paper, we propose a relation path features embedding based convolutional neural network with attention mechanism for discovering potential drugs from literature. Our method could be an auxiliary method for drug discovery, which can speed up the discovery of new drugs for the incurable diseases.

**Electronic supplementary material:**

The online version of this article (10.1186/s12911-019-0764-5) contains supplementary material, which is available to authorized users.

## Background

Despite the unprecedented advances in biotechnology, drug discovery is still a lengthy and expensive process with low rate of new therapeutic discovery [1]. Development of a new drug is estimated to take 14 years and cost approximately $1.8 billion [2]. In contrast, Literature-based Discovery (LBD) is a safe and low-cost technique that links the existing knowledge reported in unrelated literature sources for discovering new relationships [3, 4]. It generates scientific hypotheses that may help scientists, especially biomedical scientists, to accelerate the process of scientific discovery [5]. For example, Swanson first proposed the assumption that fish oil can treat Raynaud’s disease by employing LBD in 1986 [6]. Two years later, this hypothesis was verified via medical experiments [7]. Since then, a variety of automatically LBD approaches have been introduced to mine potential associations from literature, including statistics-based and co-occurrence based methods [8]. Such methods typically search for a set of intermediate terms that frequently co-occur with a source term and a target term. However, these existing LBD methods have several limitations. Statistics-based LBD relies on the number of word frequencies in cooccurrence terms, which may make it difficult to find meaningful associations for low-frequency terms [9]. Co-occurrence methods typically suffers from the imprecise meaning of such co-occurrences and logic errors [8]. Hristovski et al. introduced a semantic pattern-based LBD method which may be used to find more complex hidden associations from literature [10]. Semantic pattern-based methods could select more plausible associations between a source and a target concept. But the limitation is that the semantic patterns are manually selected and defined [11]. In addition, a number of recent LBD methods have been proposed which utilize certain graph data structures for discovering potential associations. For example, Cameron et al. proposed to automatically constructing a biological entity sub-graph through the context information of a large-scale knowledge graph, which result in a sub-graph containing complex and important information among biological entities. According to the authors, this information can promote LBD [12]. To handle large-scale knowledge graphs, random walks algorithm are often used instead of enumerating all sub-graph structures. Liu et al. proposed a method of random walks on a heterogeneous graph for drug repositioning [13]. However, due to the completely randomized mechanism, random walks are inefficient for discovering new drugs. The above method ignores the relation path features information which plays an important role in LBD. Despite these considerable advances, there is still a significant room for improvement in mining drug therapies from literature.

In this paper, we propose a convolutional neural network (CNN) model with attention mechanism method that exploits the drug-disease relation path features for drug discovery. The contributions of this paper are as follows: First, We commenced by constructing a biomedical knowledge graph with predications extracted from PubMed. Second, the path ranking algorithm (PRA) was adopted to generate drug-disease relation path features from the knowledge graph. Finally, a CNN based on attention mechanism model was trained as a drug discovery model. Then, we used the trained model to discover potential treatments for new diseases. To the best knowledge, this is the first method that employs CNN model with attention mechanism combined with relation path feature for drug discovery.

## Methods

**Fig. 1:**
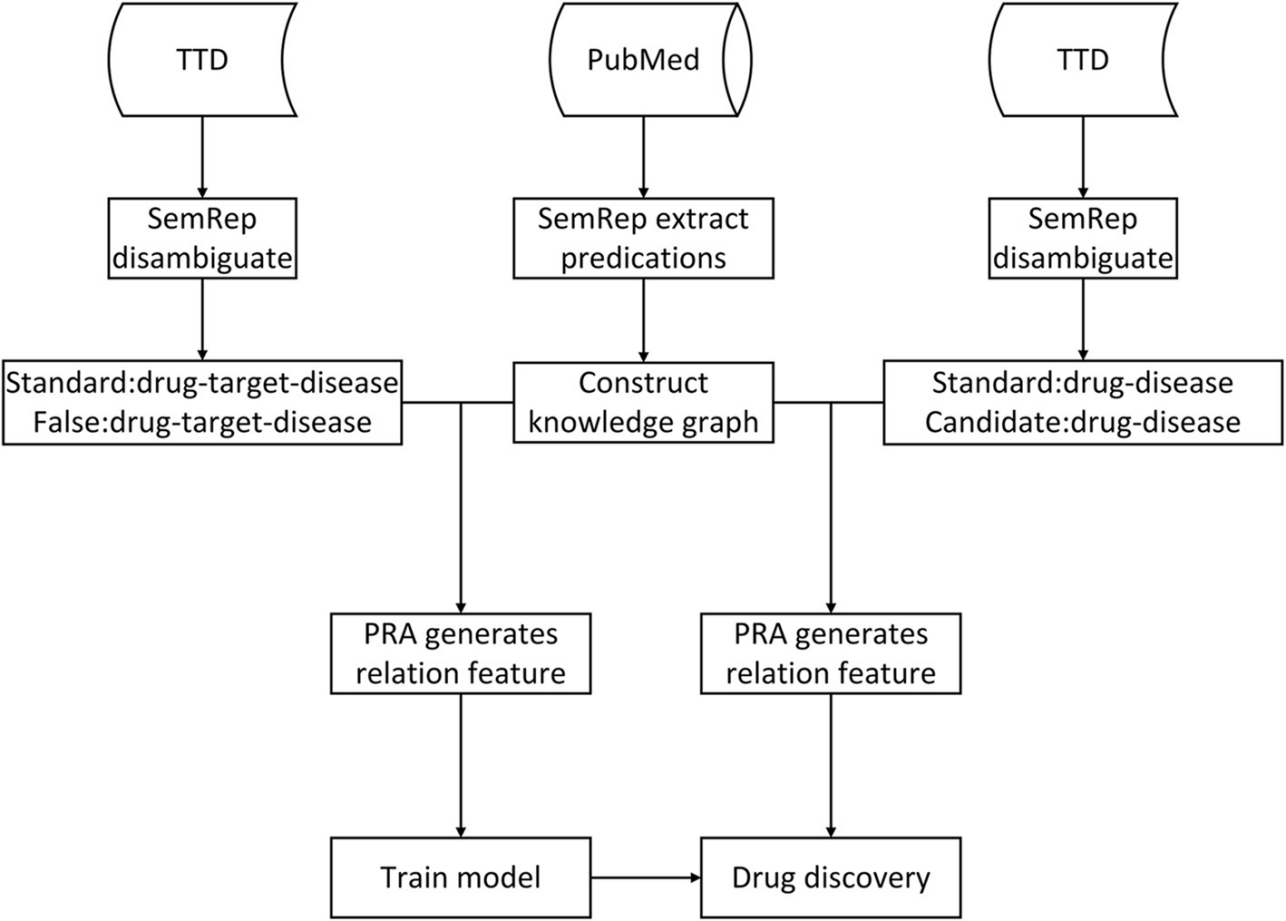
The basic process of PACNN for drug discovery

In this section, the datasets and related tools are briefly introduced. We firstly construct the biomedical knowledge graph. Then, we introduce the process of PRA obtaining data features based on the knowledge graph. After that, we use drug-disease path features to train a model, which is subsequently implemented to discover potential drugs for diseases. Finally, several metrics are introduced to measure the performance of our model and the baseline methods. Our experiment process is shown in Fig. 1.

### Datasets

#### PubMed

PubMed is a free search engine that provides biomedical paper searches and abstracts which has increased the number of entries from 17 million to more than 23 million in just eight years [14, 15]. The MEDLINE database (2013 version) was the main resource for our work.

#### Therapeutic target database

The Therapeutic Target Database (TTD) provides a wealth of information relating drugs and targets, as well as targets and diseases. The TTD produces a large number of drug-target-disease triplets that served as an appropriate resources for our work [16]. We used the standard drug-disease provided by the TTD as both training data and test data [17].

### Related tools and techniques

#### SemRep

SemRep is a Unified Medical Language System (UMLS)-based program that identifies semantic predications in biomedical texts. In this study we used SemRep to extract semantic predications from MEDLINE database [18]. Predications contain two entities and a relation, with *Triamterene*_*entity*_−*Treat*_*relation*_−*Edema*_*entity*_ being an examples of predications. Lexical ambiguity is a universal feature of natural language, similarly, there will be ambiguous words in the biomedical literature, in order to map the entities in the TTD to the knowledge graph effectively, we also used SemRep to reduce words ambiguity from TTD [19].

#### Path ranking algorithm

Path ranking algorithm (PRA) calculates the feature matrix on the pair of nodes in the graph with labeled edges. This method has strong logical reasoning ability [20]. PRA was originally used for knowledge reasoning and knowledge recommendation tasks [21, 22]. The PRA is divided into two processes, whereby all the relation types that connect a pair of nodes are enumerated in the first step. This is followed by calculating the relation path feature by performing a random walk on the graph. Once the path feature has been calculated, it can be used for any classification model, although in almost all previous applications, PRA works only used logistic regression [20]. In this paper, the relation path features generated by PRA is used to perform the drug discovery task.

#### Convolutional neural network

The convolutional neural network model has achieved remarkable results in image, speech and natural language processing (NLP) [23]. The core point of the convolutional neural network is that the convolutional layer can capture the local correlation of features, and the convolutional kernel of the convolutional layer realizes the function of receptive field. Finally, local information of the lower layer is extracted to reach a higher level through the convolution kernel [24]. For example, in a drug discovery task, information on a single relation path can determine whether a drug-disease relationship is correct or not. A certain path relation is a good indicators of drug-disease classification [25]. In this paper, we propose a CNN structure to capture relation path information for drug discovery.

#### Attention mechanism

In order to capture the most important feature of a path from a drug to a disease, we also introduce attention layer as one of the model layers. The attention mechanism was first applied to the image area and subsequently applied to the NLP, but attention mechanism has never been employed in the hypothesis discovery context. In this work, we used attention mechanism to identify important relation path features during the training in order to improve model power [26].

### Knowledge graph construction

**Fig. 2:**
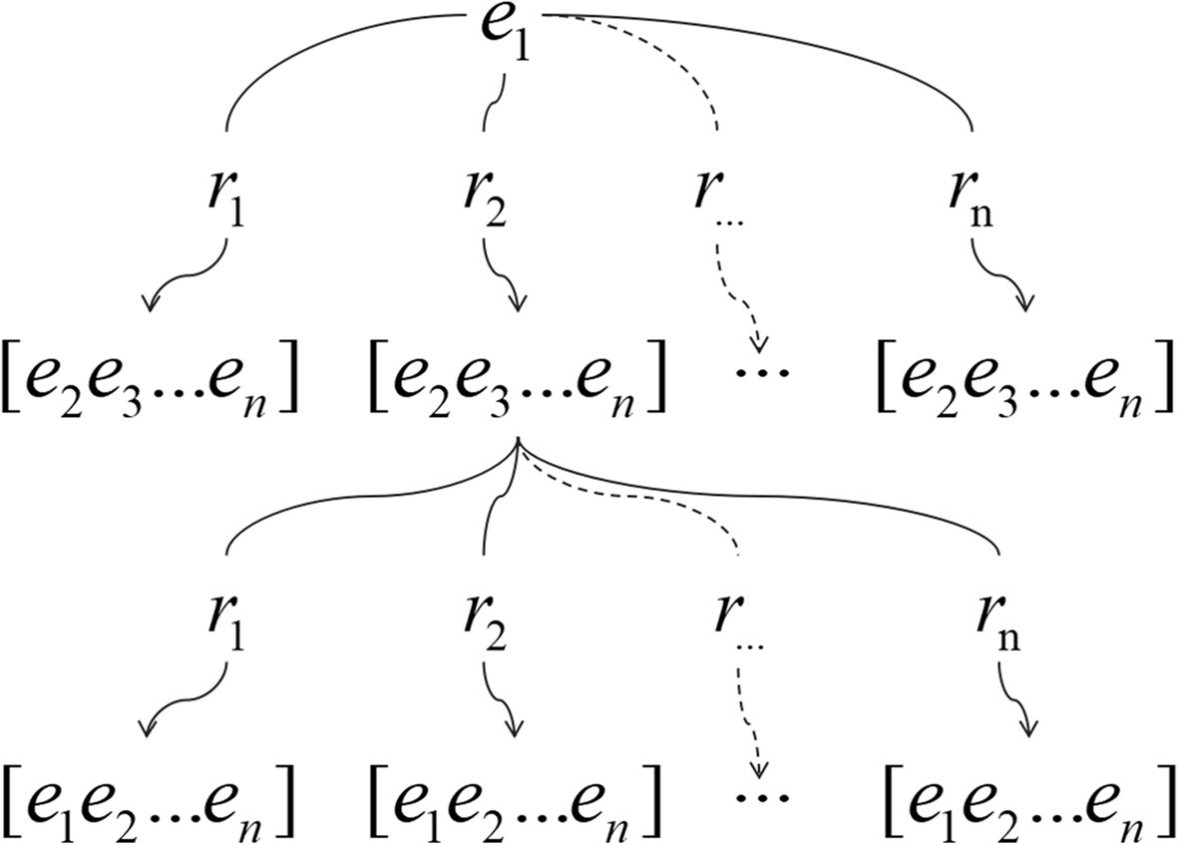
Knowledge graph structure

In general, knowledge graph (KG) comprises of different nodes and edges. In this work, we firstly obtained the predications extracted by SemRep from the biomedical text. Then, a knowledge graph was constructed by the predications. Specifically, in the KG, let *E*={*e*_1_,*e*_2_,...,*e*_*n*_} denote the nodes and *R*={*r*_1_,*r*_2_,...,*r*_*n*_} denote the edges, where *e* and *r* represent entity and relation, respectively. The KG structure (like a tree structure) is shown in Fig. 2, this is a two-level relation tree example of the KG.

### The path ranking algorithm extract drug-disease feature

Given a KG, we define P as a relation path which is only composed of relations. For example:

1. *P*_1_ : $e_{1}\ \underrightarrow {\text {inhibits}}\ e_{2} $
2. *P*_2_ : $e_{1}\ \underrightarrow {\text {inhibits}}\ e_{2} \ \underrightarrow {\text {inhibits}}\ e_{3} $
3. *P*_3_ : $e_{4}\ \underrightarrow {\text {inhibits}}\ e_{5} \ \underrightarrow {\text {inhibits}}\ e_{6} $
4. *P*_4_ : $e_{1}\ \underrightarrow {\text {inhibits}}\ e_{2} \ \underrightarrow {\text {stimulates}}\ e_{3} $
5. *P*_5_ : $e_{1}\ \underrightarrow {\text {stimulates}}\ e_{2} \ \underrightarrow {\text {inhibits}}\ e_{3} $

In the above example, *P*_2_ and *P*_3_ are the same relation path, because *P*_2_ and *P*_3_ contain the same relations although they contains different entities. In contrast, *P*_4_ and *P*_5_ are different relation paths due to the order of relations is different. Based on the above cases, we obtained 4 types of relation paths:

1. *P*_1_ : $\ \underrightarrow {\text {inhibits}}\ $
2. *P*_2_ : $\ \underrightarrow {\text {inhibits}}\ \ \underrightarrow {\text {inhibits}}\ $
3. *P*_4_ : $\ \underrightarrow {\text {inhibits}}\ \ \underrightarrow {\text {stimulates}}\ $
4. *P*_5_ : $\ \underrightarrow {\text {stimulates}}\ \ \underrightarrow {\text {inhibits}}\ $

In this work, each type of relation path is considered as a feature for training our drug discovery model. The PRA firstly enumerates all relation paths connecting two nodes. Then, the PRA recursively calculates the probability *h*_*i*,*P*(*j*)_ of the two nodes under each relation

1 $$ h_{i,P(j)}=\left\{ \begin{array}{ll} 1, & \text{if j = i}\\ 0, & \text{otherwise} \end{array}\right.  $$

Nonempty relation path $P= R_{1},R_{2},...,R_{n} \phantom {\dot {i}\!}$, and we let $P^{'}= R_{1},R_{2},...,R_{n-1}\phantom {\dot {i}\!}$

2 $$ h_{i,P(j)}= \sum_{j^{\prime} \in \text{range}(P^{\prime})} h_{i,P^{\prime}(j^{\prime})} \cdot P(j|j^{\prime};R_{n})  $$

Where $\phantom {\dot {i}\!}range\left (P^{'}\right)$ represents that under the relation *R*_*n*_, the set of entities connected to the *j*. Where $\phantom {\dot {i}\!}P\left (j|j^{'};R_{n}\right)$ is the probability of entity *j* reaching to entity $\phantom {\dot {i}\!}\it {j^{'}}$ under the relation *R*_*n*_, $P\left (j|j^{'};R_{n}\right)=\frac {R_{n}\left (j^{'},j\right)}{R_{n}\left (j^{'},*\right)}\phantom {\dot {i}\!}$, $\phantom {\dot {i}\!}R_{n}\left (j^{'},*\right)$ is the out-degree of $j^{'}\phantom {\dot {i}\!}$ under *R*, $\phantom {\dot {i}\!}R\left (j^{'},j\right)$ presents whether exists an edge connect *i* to *j* under the relation *R*.

For example, the number of relation types is *m*, and the length of the relation path is *l*. The feature length $L = \sum _{l=1}^{l} m^{l}$. Each $h_{i,P_{k}(j)}$ as a feature for *i* and *j*.

3 $$ \pi=\left[h_{i,P_{1}(j)},h_{i,P_{2}(j)},...,h_{i,P_{L}(j)}\right]  $$

Given a drug-target-disease triplets, which provides the information concerning targets and their corresponding drugs and diseases. The process of feature extraction by PRA is as follows: First, PRA obtains the a vector of relation path features between drug and target, which is denoted as *π*_*drug*−*target*_. Similarly, we then obtain the feature vector *π*_*target*−*disease*_ which denotes the relations path features between target and disease. After that, the concatenation of two feature vectors *π*_*drug*−*target*−*disease*_ is considered as the features for the given drug-target-disease triplet. Therefore, for each drug-target-disease, a training data (*π*_*train*_,*y*) is constructed, where *y* is a boolean variable indicating whether the case is positive.

### Training model

**Fig. 3:**
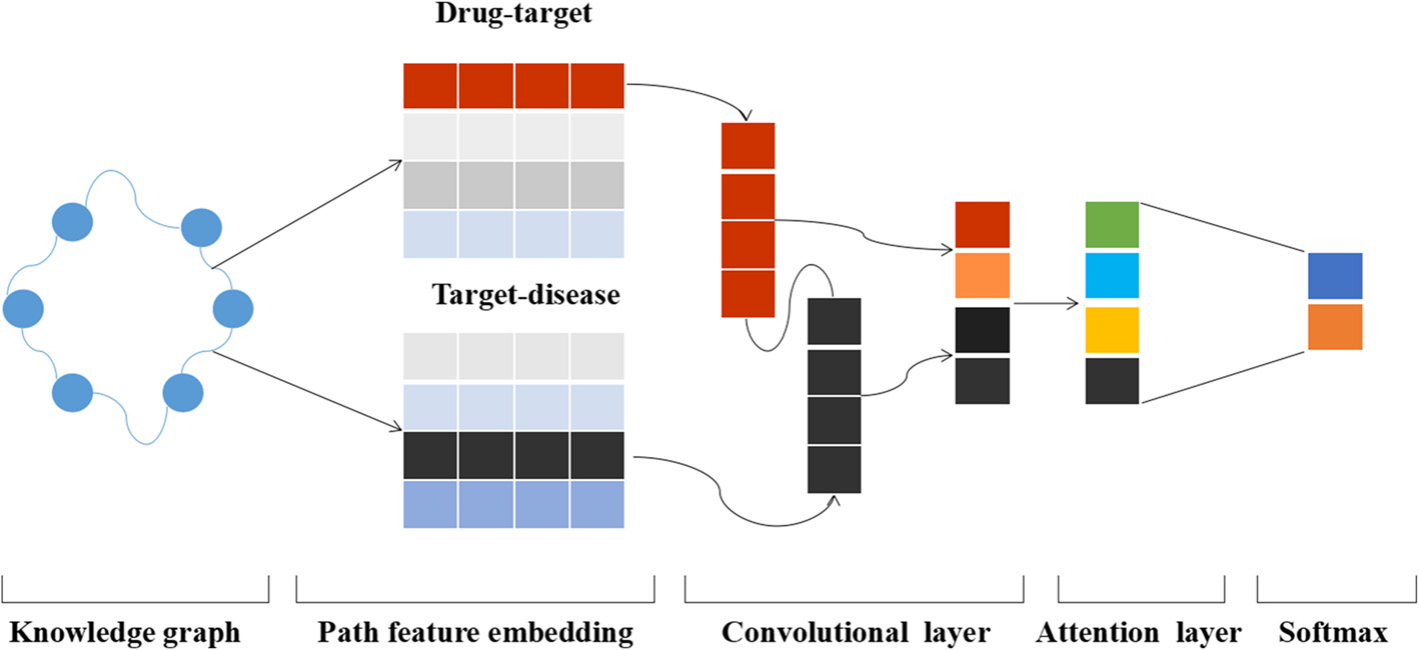
The architecture of neural networks for drug discovery

This work employs CNN based on attention mechanism as the basic model. The neural network model structure is shown in Fig. 3, our model is trained to predict conditional probability *P*(*y*|*π*;*θ*). where *θ* are parameters of our model for the relation path features. Let *p*_*i*_ be the path feature of drug-target-disease, a set of path feature represents as

4 $$ p_{k:n}=p_{1} \oplus p_{2} \oplus... \oplus p_{n}  $$

Where ⊕ indicate concatenation, the CNN sliding window size is *k*, the vectors became as follows after falling into sliding windows,

5 $$ P_{k}=\left[ p_{k},p_{k+1},...,p_{k+m-1} \right]  $$

Combine the window with the filter to convolutional operation for obtaining new features.

6 $$ Y_{k}=f(P_{k} \odot W + b)  $$

Where *f* is a nonlinear activation function, in this experiment, we utilize the ReLU activation function; ⊙ is the convolution operator; *W* is the convolution kernel; *b* is bias term. Then, this operation makes a feature map, such as

7 $$ C=\left[ c_{1},c_{2},...,c_{n-k+1} \right]  $$

Where *C*∈*R*^*n*−*k*+1^, we employ max pooling layer on the feature map which keeps the most important feature for each map. Not all relation path features contribute equally to the representation of the drug-disease relation. Here, we employ attention mechanism to extract relation path that are important to the association of the drug-disease [27]. Due to the drug-disease relation types are too many, three-layer CNN are used to compress the number of relation types. We get *h*_*it*_ from CNN max pooling layer, *t*∈[0,*L*], *L* is the number of relation types after compression, the attention layer calculations formula is as follows

8 $$ u_{it}=tanh\left(W_{w} h_{it} + b_{w}\right)  $$

Where *W*_*w*_ is the attention layer weights matrix, *b*_*w*_ is bias term.

9 $$ \alpha_{it} = \frac{exp\left({u_{it}}^{T} u_{s}\right)}{\sum_{t} exp\left({u_{it}}^{T} u_{s}\right)}  $$

10 $$ s_{i}={\sum_{t} {\alpha_{it} h_{it}}}  $$

Then we measure the significance of the relation path feature, weight is obtained by calculating the similarity of *u*_*it*_ to the relation context vector *u*_*s*_, and the softmax method generate a normalized significance weight *α*_*it*_. After that, we obtain the relation path vector *s*_*i*_ through a weighted sum of the relation path based on the weights. In the end of the neural networks, we combine with fully connected layer and softmax layer, the softmax layer classify the drug-disease feature into two categories and give the probability for each category. While training the model, tune model parameters by gradient descent and back propagation.

### Implementation for drug discovery

In order to determine the effectiveness of drug treatment for a particular disease, whereby all drugs may become candidates for the discovery of drugs that can treat diseases. Every *drug*_*candidate*_−*target*_*candidate*_−*disease* relation path feature as *π*_*candidate*_. A candidate drug produce many sets of relation paths features as *¶*_*candidate*_=[*π*_1_,*π*_2_,...,*π*_*n*_] by aforementioned method, whereby the candidate drug score is defined below

11 $$ score\left({drug}_{candidate}\right)=\frac{1} {\eta} \sum sorted(d(y \geq0.5|\P,\theta))[:\eta]  $$

Where *d* is discriminate methods with parameter *θ*. Our method gives every case positive category probability, and all cases are ranked from large to small according to the probability *y*. Thus, all cases probability ranked in the top *η%* are selected. Finally, the candidate drugs are ranked according to their scores.

### Random walk baseline method

Here, we compare our method with some baseline which use the Random Walk (RW), the RW generates Markov chains on a directed graph and will reach a equilibirum state in a certain number of steps [28]. We define a state transition probability matrix *P*, and *P*_*ij*_ indicates the probability of the two-node connection on the graph [29].

12 $$ P_{i j}=\left\{ \begin{array}{ll} 1/d_{i}, & j \in Adj(i)\\ 0, & j\notin Adj(i) \end{array}\right.  $$

Where node *i* out-degree is *d*_*i*_, *Adj*(*i*) is the set of adjacent nodes of *i*, $\sum _{j=1}^{N} P_{i j}=1$, within one step, the probability of a node jumping to all neighboring nodes are the same. We define *M*=(*P*_*ij*_)*i*,*j*∈*N* as Markov start chain [30]. Matrix transfer rules are as follows

13 $$ M^{t+1}=P^{T}M^{t}  $$

**Fig. 4:**
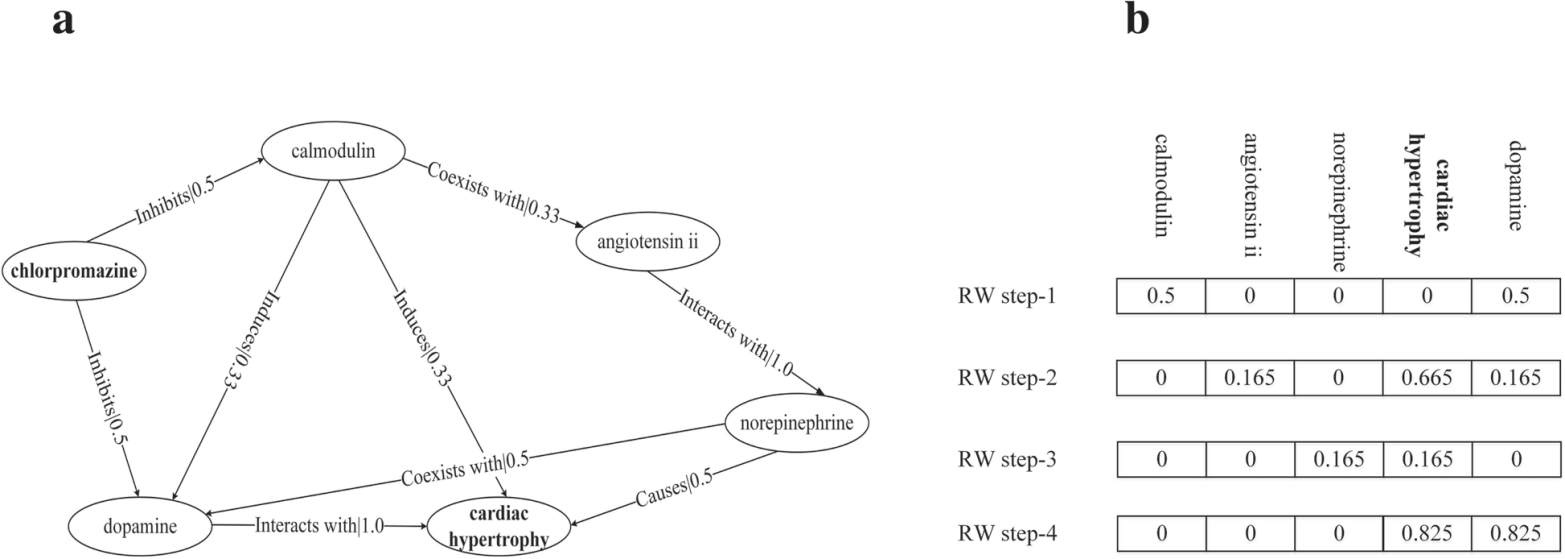
Random Walk for drug discovery **a** is semantic graph, **b** is the results of the random walks

In the matrix *M*^*t*^, the $M^{t}_{i j}$ is the probability of starting node *i* reaches node *j* in *t* steps. Figure. 4 shows how the drug ‘chlorpromazine’ random walks to the disease ‘cardiachypertrophy’. Figure. 4a is a semantic graph with weights. Figure. 4b shows the results of random walks with different steps. In this example, ‘chlorpromazine’ as the initail node can not reach the ‘cardiachypertrophy’ in step-1, thus, the score of candidate drug ‘chlorpromazine’ is 0. When the number of steps exceeds 2, the ‘chlorpromazine’ can reach the ‘cardiachypertrophy’. The 0.665, 0.165, and 0.0825 represent the scores of the ‘chlorpromazine’ treating the ‘cardiachypertrophy’ in different steps, respectively. RW calculates the candidate drugs score for the disease, and the candidate drugs are ranked according to the score.

## Results

In this section, we first introduce the details of the KG and the training data, followed by several metrics used to measure the performance of our method. Finally, we present several cases to show the ability of our model for discovering potential drug.

### Data preparation

In this work, we extracted 1,714 drug-target-disease cases from TTD as golden standard cases, see Additional file 1. It is necessary to ensure that the nodes of triplet exist in the KG, and set the path relation length *l* to 2 and relation types *m* to 52, each data feature length is (52+52^2^)+(52+52^2^), KG materials shown in Table 1. The number of false samples is the same as that of positive samples, which is randomly selected where they not exist in TTD.

**Table 1:** Corpus materials statistics

KG materials	Number
PubMed abstracts	22,769,789
Predications	39,133,975
Entities	658,151
Relation types	52

### Implementation details

We implemented our methods using the Scikit-learn and Keras library [31, 32]. We used softmax for drug-disease relation classifiers, the filter numbers of the three CNNs were 128, the number of neurons of attention layer is 128 and Softmax layer is 256, the mini-batch size was set as 52, the model was trained for at most 12 epochs.

### Ten-fold cross-validation

We conducted ten-fold cross validation to evaluate the performance of our method. The data set was divided into ten parts, and nine of which were taken as the training data and one was used as the test data. Each test will result in a corresponding predicted score. The average of the predicted score of the 10 results is used as an estimate of the algorithm performance. The experiment uses the precision rate (P), recall rate (R), and f-score (F) to evaluate the model effectiveness. The specific calculation formula is as follows

14 $$ P=\frac{N_{TP}}{N_{TP} + N_{FP}}  $$

15 $$ R=\frac{N_{TP}}{N_{TP} + N_{FN}}  $$

16 $$ F=\frac{2P\cdot R}{P + R}  $$

Define a data set: The number of samples is represented by *N*, *N*_*TP*_ represents positive samples and prediction is positive samples too; *N*_*FN*_ represents positive samples but prediction is negative samples. *N*_*TN*_ represents negative samples and prediction is negative samples. *N*_*FP*_ is actually a negative sample, but the prediction is a positive sample.

Table 2 shows the results of a comparison between our PACNN model and other state-of-the-art methods.

**Table 2:** The performance of different model

Methods	Precision(%)	Recall(%)	F-score (%)
SVM	78.55	71.69	69.73
RF	84.73	84.49	84.38
LR	87.00	86.30	86.14
CNN	90.81	90.82	90.76
PACNN	91.50	91.50	91.46

From Table 2 we can see that PACNN model outperforms Support Vector Machine, Random Forest, Logistic Regression and Convolutional Neural Network. The input features of the machine learning model are the same as the PACNN, and the parameters of machine learning methods are set according to the best experimental results. The PACNN model and the CNN model have the same parameter settings, except that the PACNN adds an attention layer behind the CNN. We argue that PACNN model classify drug-disease more effectively. It can not only extract more abundant features with CNN from a drug-disease, but also capture important path features with the attention layer. In order to verify that the relation path feature is more suitable for our proposed model, we use two alternatives to verify the validity of the feature. In one method, we convert the path non-zero feature to a random value between 0 and 1, while in the other, we convert all non-zero features to 1. Our proposed relation path feature has greatly improved, comparing to the other features. Table 3 shows the results, indicating that the PRA not only keeps the inference mode, but the relation path feature preserves the significant information about drug-disease.

**Table 3:** The PACNN model with different embedding feature

Methods	Precision(%)	Recall(%)	F-score(%)
PACNN-random	83.21	82.68	82.53
PACNN-one	86.43	85.79	85.56
PACNN-pra	91.50	91.50	91.46

### Drug rediscovery

To verify the ability of the model to discover new drugs for known disease, we selected 300 gold standard drug-disease from TTD, see Additional file 2, while there are 96 drug-disease directly connected in the KG, resulting in 204 cases. For a new disease, we randomly selected 100 candidate drugs as potential drugs, while also including standard drugs for treating diseases. Since the mechanism by which the drug acts on the disease is not clear, in order to ensure that the drug candidate can be linked to the disease under the corresponding target, we selected 3,564 targets from TTD as candidate targets. The validation criteria for drug discovery experiments are candidate drug score mean rank and hit@10, indicating that the candidate drug score rank in top 10. In fact, the scores of candidate drugs are ranked in top, indicating that the candidate drug is closer to the real therapeutic drug. If a drug for treating the disease is not found, the corresponding drug score and mean rank are not considered in the total number.

**Fig. 5:**
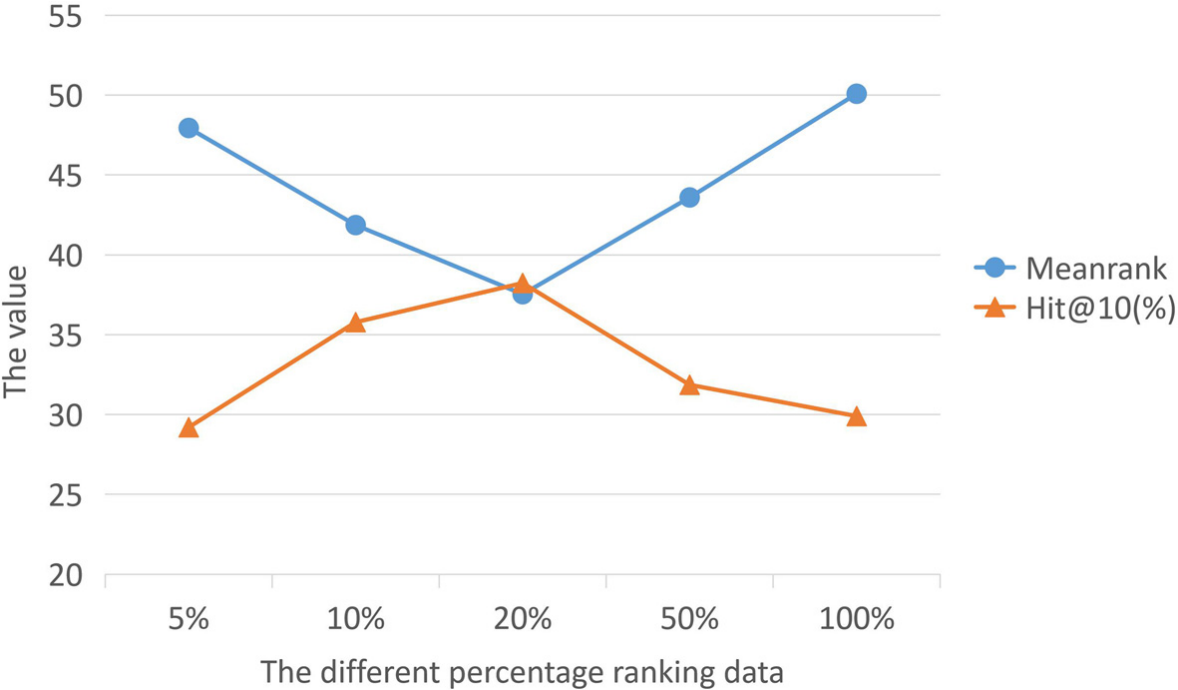
The performance of different percentage data

NRWRH and TP-NRWRH are additional baseline methods, both of which are drug repositioning methods employing random walks with heterogeneous network. The difference is that TP-NRWRH uses two-pass random walks [13, 33]. For the drug-disease score and ranking, this work set the RW maximum steps size is 5, and the parameters of other baseline methods are set the recommended settings in their experiment. In Table 4 methods column, RW-2 represents random walks algorithm step is 2, if a drug reach to a disease in 1 step, it indicates that the drug has a therapeutic effect on the disease. The ‘Not Found’ column indicates that the current method cannot find the known drug number. Table 4 shows that, if the number of the walk steps exceeds 3, all drugs can be found by the RW. This means that all drugs and diseases are connected in at least 3 steps in the KG. On the other hand, 20 and 13 drugs are not found by the NRWRH and TP-NRWRH, respectively. Although the number of walking steps of these two methods is 3, due to they use restart random walks on the heterogeneous network, the drug can not reach the disease accurately. In Table 4, the best result of ‘Mean Ranking’ column RW method is 55.26 by the RW-2. When the steps increase, the more candidate drug will be found, this will increase the mean ranking of the RW. NRWRH and TP-NRWRH outperform the RW, as the random walks are based on specific a heterogeneous network. In addition, in the column ‘Hits@10’, the performance of NRWRH and TP-NRWRH is still better than RW. We see that, when the steps increase, the mean ranking and hit@10 score approach the steady state. Finally, from Table 4 we see that PACNN shows the best performance on two tests, as the ‘Mean Ranking’ is 37.53 and ‘Hit@10’ is 38.23%. Compared with random walk based methods, our method not only finds all candidate drugs but produces the best results. Additionally, we vary the settings of *η* to see how different percentage data affects the results. A set of scores are produced by a candidate drug and we would normally select the greatest among these values for the candidate drug. Due to the same scores are produced in 100 candidate drugs, while the number of intermediate candidate targets for each candidate drug and disease is uncertain. Thus, we chose the average of the different proportions of a set of scores as the final candidate drug score. We set *η* as top 5%, 10%, 20%, 50%, and 100% respectively. In addition, when the model prediction case is negative, we filter out data with the probability less than 0.5. In Fig. 5, we can see that when the data reached top 20%, the best results were achieved in both tests.

**Table 4:** Drug rediscovery performance

Methods	Not found	Mean ranking	Hit@10(%)
RW-2	33	55.26	17.54
RW-3	0	63.28	11.76
RW-4	0	64.04	10.78
RW-5	0	64.57	10.78
NRWRH	20	58.14	21.19
TP-NRWRH	13	41.54	29.31
Our method	0	37.53	38.23

### Case study

To demonstrate the capabilities of our model, we show 12 samples that ranked in top 10. PACNN can predict candidate drug that is reported by TTD as capable of curing a disease. From Table 5 we can see that the drug ‘Typherix’ treats disease ‘Salmonella infection’, it ranked 1st as the candidate drug and TTD provides a research phase in the treatment of diseases at the column ‘Drug Status’.

**Table 5:** Case study: rediscover known drugs for diseases

Drug	Disease	Rank	Drug status
Typherix	Salmonella infection	1	Approved
INS-1	Metabolic disease	2	Approved
Triamterene	Edema	5	Approved
Triamterene	Congestive heart failure	3	Approved
Anapsos	Atopic dermatitis	3	Approved
Brevenal	Cystic fibrosis	2	Investigative
Diphencyprone	Alopecia	7	Phase 2
ECFCs	Cardiovascular disorder	7	Investigative
Pneumovax 23	Otitis media	1	Approved
Mesoglycan	Cerebrovascular disorders	1	Approved
LASSBio-294	Hypertension	6	Investigative
Simethicone	Dyspepsia	9	Approved

## Discussion

According to the experimental results, our proposed model can effectively carry out the LBD task. This is the first attempt to employ PRA and attention mechanism for LBD. However, there are several limitations affecting our works. First, the data set used to train the model is small, and this will lead to weaker generalization. Thus, it would be useful to combine other drug-disease databases, such as Comparative Toxicogenomics Database (CTD) and Drugbank for addressing this limitation [34, 35]. In order to maintain the great connectivity of the KG, we have chosen all the predications as graph components. Since a predication may be erroneous, this will reduce the efficiency of our model. This limitation can be eliminated by improving the NLP technology. Another limitation is that PACNN needs to obtain all the relation paths between drugs and diseases. When the size of the knowledge base is large, it is difficult for our method to produce a more complex relation path. When the PRA is faced with a larger knowledge base, the computational efficiency will be greatly reduced, which must also be solved in future studies.

## Conclusion

In this study, we presented a relation path features embedding based CNN with attention mechanism for discovering potential drugs from literature. Relation path feature embedding proved to be effective for capturing the association about drug-disease, thus we utilized PRA to get drug-disease relation path feature. Compared with other methods, the CNN based on attention mechanism can better identify the important relation feature of drug-disease, so that new drugs can be accurately discovered. Our method could be an auxiliary method for drug discovery, which can speed up the discovery of new drugs for the incurable diseases.

For the future work, we plan to explore an efficient path walk algorithm that is better adapted to large knowledge base. We are interested in applying our model to literature mining in other fields, such as economics. We will continue to explore the innovation and application of deep learning and machine learning on LBD tasks.

## Additional files

Additional file 1 The 1714 drug-target-disease cases which are extracted from Therapeutic Target Database(TTD) as true cases for constructing training data. (TXT 87 kb)

Additional file 2 The gold standard drug-disease cases extracted from TTD. There are 300 drug-disease case are selected from TTD as gold standard test data for drug rediscovery. (TXT 11 kb)

## Abbreviations

CNN: Convolutional neural network
LBD: Literature-based discovery
LR: Logistic regression
NLP: Natural language processing
RF: Random forest
SVM: Support vector machine
TTD: Therapeutic target database
